# Multiple geriatric syndromes in community-dwelling older adults in China

**DOI:** 10.1038/s41598-024-54254-y

**Published:** 2024-02-12

**Authors:** Ling-Ying Wang, Zi-yi Hu, Hong-xiu Chen, Meng-lin Tang, Xiu-ying Hu

**Affiliations:** 1 Innovation Center of Nursing Research and Nursing Key Laboratory of Sichuan Province, West China Hospital, School of Nursing, Sichuan University, Chengdu, 610041 China; 2 Critical Care Medicine Department, West China Hospital, Sichuan University, Chengdu, 610041 China; 3 Nursing Department, West China Hospital, Sichuan University/West China School of Nursing, Sichuan University, Chengdu, 610041 China

**Keywords:** Geriatric syndrome, Older adults, Polypharmacy, Malnutrition, Frail, Cognitive, Geriatrics, Health services, Quality of life

## Abstract

This study aims to assess the prevalence of geriatric syndromes and identify factors associated with multiple geriatric syndromes in community-dwelling older adults in China. We utilized a convenience sampling method to recruit older adults and from one rural and one urban community in Chengdu, China, from October 2022 to March 2023. A total of 706 older adults aged 60 years or older were included. Ten geriatric syndromes were investigated including two mental disorders: depressive symptoms, cognitive impairment; and eight somatic disorders: pain, falls, sleep disturbance, constipation, polypharmacy, multimorbidity, malnutrition and frailty. Multiple geriatric syndromes were defined as an individual having two or more geriatric syndromes. The data obtained were analysed using descriptive statistics. The independent risk factors for multiple geriatric syndromes were assessed using a logistic regression model. This study found that 90.5% of the participants had at least one geriatric syndrome, with 72.8% experiencing multiple geriatric syndromes. The top four geriatric syndromes in our study were polypharmacy (58.5%), malnutrition/at risk of malnutrition (43.1%), multimorbidity (42.1%), and frailty/prefrailty (34.3%). Of the older adults, 368(52.1%) had only somatic disorders, 18(2.5%) had only mental disorders and 253 (35.8%) had somatic-mental disorders. According to the logistic regression analysis, residence, age, marriage, BMI, and self-related health were significantly associated with multiple geriatric syndromes among older adults. This study highlights that multiple geriatric syndromes are prevalent among community-dwelling older adults in China, and underscores the significance of certain demographic factors in their occurrence. Future longitudinal studies are needed to establish the temporal relationship between multiple geriatric syndromes and these demographic factors, as well as to explore causal relationships and effective prevention strategies for geriatric syndrome.

## Introduction

Geriatric syndrome refers to a group of diverse health conditions that are commonly observed in older adults and do not fall into distinct disease categories^1,2^. Many of the most prevalent conditions managed by geriatricians, including delirium, falls, frailty, dizziness, syncope and urinary incontinence, are classified as geriatric syndromes^3^. Geriatric syndromes arise from dysfunction across multisystem, often making them challenging to treat effectively. If geriatric syndromes are left untreated, the activity of daily living (ADL) will decline^4^, increasing the risk of adverse outcomes such as physical disability, poor quality of life, and death^5,6^. While medications may have limited effectiveness in treat geriatric syndromes, well-planned care can be instrumental in preventing further progression^4^. Previous studies have identified several demographic factors that are associated with the development of geriatric syndromes, including age, education level, self-rated health, chronic diseases, alcohol and drug use problems^7–9^.

In recent years, geriatric syndromes have attracted increasing attention. A nationwide study involving 779 older Americans reported that 82% exhibited one or more geriatric syndromes^10^. In another study, 1705 community-dwelling men aged 70 and over in Sydney reported that conditions such as poor mobility, recurrent falls, urinary incontinence, dementia and frailty phenotype were relatively uncommon (less than 10%) in men in their 70 s, but the prevalence of each of these conditions exceeded 10% in men aged 85–89^11^. As of 2020, the number of older adults (aged > 60 years) in China reached 264 million^12^, previous studies have primarily focus on the prevalence and risk factors of individual conditions such as frailty^13^, depression^14^, sarcopenia^15^, cognitive risk^16^, nutritional risk^17^ and so on in Chinese older adults, respectively. Few studies focus on multiple geriatric syndromes and related factors.

It is worth highlighting that certain geriatric syndrome symptoms are particularly prevalent and greatly impacted by long-term care, such as, mobility disorders (remain secluded, or bedridden), falls, incontinence, and cognitive impairment and the COVID-19 pandemic has further exacerbated the risks faced by the geriatric population^18^. Addressing these issues promptly through early intervention is essential and should be a focal point in geriatric care strategies^19^. A systematic review has revealed that sarcopenia is associated with adverse outcomes, including elevated mortality rates, functional decline, increased fall risk, and increased hospitalizations, making it a substantial public health burden within the geriatric population^20^. The medical burden in the older population over 90 years could influence general health status significantly, with a high prevalence of chronic diseases and geriatric syndromes^21^. Furthermore, the limited time that primary care physicians typically spend with each patient^22^, is often insufficient for comprehensively assessing the complex needs of a geriatric patient, highlighting the need for improved approaches to care for this vulnerable population.

The objective of screening is to utilize an interdisciplinary team to identify and treat geriatric syndromes in order to prevent further functional decline or morbidity. This allows for the implementation of comprehensive and targeted interventions. Awareness and recognition of geriatric syndromes among older individuals can enhance patient-centered care and significantly improve healthcare outcomes. Therefore, the present study aimed to assess the prevalence of geriatric syndrome and identify multiple geriatric syndrome-related factors among older adults in China.

## Methods

### Sampling and recruitment

We utilized a convenience sampling method to recruit older adults in Chengdu, China, from October 2022 to March 2023. We selected one rural and one urban community based on geographical convenience. The study targeted older adults living in the community, and an information letter was provided to all eligible individuals. The research team collected data after obtaining their consent and signatures on the study's informed consent form. The study protocol was approved by the Ethics Committee of West China Hospital, Sichuan University in 2022 (Ethics No. 861). All methods were performed in accordance with the relevant guidelines and regulations.

During the study period, all older adults who met the following criteria were included: (i) age ≥ 60 years and (ii) willingness to collaborate with the researchers after the purpose of the research was explained. The exclusion criteria were as follows: mental disorders (Alzheimer's disease, Schizophrenia), severe and end-stage diseases (expected life < 12 months, that with an established life-limiting condition or in receipt of end of life palliative care services^23^). Convenience sampling was employed and the sample size was calculated using the following formula^24^:

$$n={Z}^{2}\times \frac{p\times q}{{e}^{2}}$$

where n = minimum required sample size. Z = 1.96 at 95% Confidence Interval (CI). *P* = prevalence of multiple geriatric syndrome among the elderly, 40.2%^25^. q = 1-p. e = margin of error, 5%

Based on the calculated sample size formula, a minimum of 369 participants was required to ensure a representative sample. The study ultimately included 706 older adults, thus enhancing the data's robustness and the study's generalizability.

### Procedure and data collection

The research team was composed of research nurses and the first author. Before the start of the formal data collection, the first author provided training and education to 2 research nurses on how to complete the questionnaire accurately and consistently. There was an assessment at the end of the training, and the 2 nurses who were employed as the research nurses in the study had similar assessment scores, ages, years of experience, levels of education, and job titles. Finally, these 2 nurses were able to complete the questionnaire with unified introduction and questioning method during the formal investigation to ensure consistency among investigators and reduce data collection errors caused by different questioning methods among investigators. The interview was conducted directly with older adults face to face or by telephone.

#### Sociodemographic variables

We designed a questionnaire to collect sociodemographic data from the participants. The variables included age, sex, height, weight, marital status, resident, educational level, and self-related health.

For the classification of educational level, we defined three categories: low (with less than 9 years of education), middle (with 9 to 12 years of education), high ((with more than 12 years of education). To calculate the Body Mass Index (BMI), we used the following formula: BMI = weight (in kg)/height^2^ (in m^2^). Based on the calculated BMI, the participants were divided into different categories: severely underweight (BMI less than 16.5 kg/m^2^), underweight (BMI under 18.5 kg/m^2^), normal weight (BMI greater than or equal to 18.5 to 23 kg/m^2^), overweight (BMI between 23 and 24.9 kg/m^2^), and obese (BMI greater than 25 kg/m^2^) groups^26^.

#### Geriatric syndromes

The geriatric syndromes included two mental disorders (depressive symptoms, cognitive impairment), as well as eight somatic disorders (pain, falls, sleep disturbance, constipation, polypharmacy, multimorbidity, malnutrition, and frailty). These geriatric syndromes were chosen because of their high prevalence in older adults^1–3^. The specific measurement scales/tools and methods for each geriatric syndrome are detailed in Table 1. The study defined multiple geriatric syndromes as the coexistence of two or more geriatric syndromes.

**Table 1:** The measurement scales/tools and methods for geriatric syndromes in our study.

Geriatric syndrome	Scale/tool	Method
**Mental disorders**		
Depressive symptoms	Patient Health Questionnaire-9 (PHQ-9) in Chinese, which consists of 9 questions that are based on the 9 DSM-IV criteria for a major depressive disorder, including anhedonia, depressed mood, sleep disturbance, fatigue, appetite changes, low self-esteem, concentration problems, psychomotor disturbances, and suicidal ideation^27^	Depressive symptoms were assessed by the the scores for each PHQ-9 item range from 0 (not at all) to 1 (several days), 2 (more than half of the days), and 3 (nearly every day). Furthermore, the PHQ-9 is validated as a depressive symptom severity measure (5–9: mild depression, 10–14: moderate depression, 15–19: moderately severe depression, and 20–27: severe depression)^28^. In our study, a score > 5 was used as the cut-off
Cognitive impairment	the Mini-Mental State Examination (MMSE) in Chinese, which is a 30-point questionnaire used extensively in clinical and research settings to measure cognitive impairment, including simple tasks in a number of areas: the test of time and place, the repeating lists of words, arithmetic such as serial subtractions of seven, language use and comprehension, and basic motor skills^29^	Mild cognitive impairment (MCI) was identified using education-specific cut-off points of total scores of MMSE: MMSE ≤ 19 for illiterate individuals, ≤ 22 for participants with elementary school education, and ≤ 26 for those with middle school education and above^30^
**Somatic disorders**		
Pain	Wong-Baker FACES Scale (WBS) to measure pain level	A score > 0 was used as the cut-off^31^
Fall	Record the number of falls in the past year	Having at least one fall was classified as being a faller
Sleep disturbance	Participants were asked about the number of hours they actually slept on a typical night in the past month	Five or fewer hours was used as the cut-off^2,32,33^
Constipation	Rome-III criteria to measure constipation	In the absence of functional constipation without laxative use, two or more of the criteria were at least two months prior to diagnosis: 1) two or fewer defecations in the toilet per week, 2) at least one episode of fecal incontinence per week, 3) history of retentive posturing or excessive volitional stool retention at least once a week, 4) history of painful or hard bowel movement at least once a week, 5) presence of a large fecal mass in the rectum at least once a week, and 6) history of large diameter stools that may obstruct the toilet at least once a week^34^
Polypharmacy	–	Polypharmacy was defined as the current use of five or more prescription and over-the-counter medications^35^
Multimorbidity	–	Multimorbidity was defined as an individual having two or more current chronic diseases^36^
Malnutrition	MNA-SF, a six-item instrument with a score range of 0 to 14 points^37^. The MNA-SF has been validated and shows good specificity and sensitivity for the diagnosis of malnutrition, especially in older adults^38,39^	For the purpose of our study, participants were categorized into a group with normal nutritional status (12–14 points), individuals at risk of malnutrition (8–11 points), or malnourished individuals (0–7 points)^40^. The MNA-SF has been validated among elderly individuals in China and has extraordinary test characteristics^41,42^
Frailty	FRAIL scale, which includes five self-reported questions: Do you feel fatigued? Can you climb a flight of stairs? Can you walk a block without stopping? Do you follow up or treat more than 5 diseases? In the last 6 months, have you unintentionally lost 5% or more of your body weight?	The score ranges from 0 to 5 points, and each component of the assessment is worth one point. Individuals were classified as “nonfrail” (score 0), “prefrail” (score 1–2), and “frail” (score 3–5)^43^

The diagnosis of elderly syndrome is evaluated by the research nurses using the measurement scales/tools, and a general practitioner makes a diagnosis based on the evaluation results (Table 1). Older adults who had one or more somatic disorders and none mental disorders were divided into somatic disorders group; older adults who had one or two mental disorders and none somatic disorders were divided into mental disorders group; older adults who had both one or two mental disorders and one or more somatic disorders were divided into somatic-mental disorders group.

### Statistical analysis

All data were categorical variables. To describe the demographic information, descriptive statistics were presented as counts and percentages. The Statistical Package for the Social Sciences (version 24.0) was used to enter and analyse the counts and percentages. The chi-squared test was utilized to evaluate the association between demographic information and self-related health with the presence of multiple geriatric syndromes. Statistical significance was set at *P* < 0.05 (two-tailed). To enhence clinical interpretability, adjacent groups with similar clinical significance and a sample size of less than 5% within the group were merged.

A Logistic regression model was conducted using the multiple geriatric syndromes as dependent variables and other factors as independent variables. The B, SE (standard error), Wald, 95% CI, and P values were reported. The results of the model were presented as odds ratios (ORs) with 95% confidence intervals (CIs).

## Results

In our study, 706 older adults were evaluated, with an average age of 70.75 ± 7.39 years. The majority (56.5%) lived in urban community, 42.9% were female, 21.7% had a high education level, 88.5% were married, 36.3% had normal weight, and 75.2% reported self-related good health.

Among the participants, 67(9.5%) did not have any geriatric syndromes. The most prevalent geriatric syndromes were polypharmacy (58.5%) and malnutrition/ at risk of malnutrition (43.0%). Regarding the distribution of geriatric syndromes, 368(52.1%) older adults had only somatic disorders, 18(2.5%) had only mental disorders and 253(35.8%) of them had somatic-mental disorders. Table 2 provides the numbers of geriatric syndromes and percentages of associated conditions. Among the 125 participants with one geriatric syndrome, polypharmacy was the most common condition. Among those 514 participants with multiple geriatric syndromes (≥ 2 geriatric syndromes), the most prevalent syndromes were polypharmacy (73%) and multimorbidity (56%).

**Table 2:** Number of geriatric syndromes and percentages of associated conditions.

Geriatric syndromes	Total	1	≥ 2
Numbers of participants	706	125	514
Polypharmacy	413 (58.5)	40 (0.32)	373 (0.73)
Malnutrition/at risk of malnutrition	304 (43.1)	25 (0.2)	279 (0.54)
Multimorbidity	297 (42.1)	10 (0.08)	287 (0.56)
Frail/pre-frail	242 (34.2)	4 (0.03)	238 (0.46)
Cognitive impairment	226 (32.0)	13 (0.10)	213 (0.41)
Pain	150 (21.2)	18 (0.14)	132 (0.26)
Depressive symptoms	92 (13.0)	5 (0.04)	87 (0.17)
Constipation	88 (12.5)	4 (0.03)	84 (0.16)
Sleep disturbance	80 (11.3)	6 (0.05)	74 (0.14)
Fall	36 (5.1)	0	36 (0.07)

Table 3 compared demographic factors that were significant associated with multiple geriatric syndromes in older adults in univariate analyses. Our results indicated that there were significant differences in the occurrence of multiple geriatric syndromes between different residents (*P* = 0.001). Specifically, 67.9% of older adults living in urban communities and 79.2% of those living in rural communities had multiple geriatric syndromes. Other significant factors included age (*P* < 0.001), sex (*P* = 0.002), marriage (*P* < 0.001), BMI (*P* < 0.001), and self-related health (*P* < 0.001).

**Table 3:** Results of univariate analyses of associations between demographic factors and multiple geriatric syndromes in older adults.

Variable	Multiple geriatric syndromes		χ^2^	*P* value
	No	Yes		
Resident			11.058	0.001
Urban	128 (32.1)	271 (67.9)		
Rural	64 (20.8)	243 (79.2)		
Age(years)			43.547	< 0.001
60–69	132 (38.6)	210 (61.4)		
≥ 70	60 (16.5)	304 (83.5)		
Sex			9.889	0.002
Male	128 (31.8)	275 (68.2)		
Female	64 (21.1)	239 (78.9)		
Education level			5.410	0.067
Low	110 (25.2)	326 (74.8)		
Middle	36 (25.7)	104 (74.3)		
High	46 (35.4)	84 (64.6)		
Marriage			20.423	< 0.001
Married	187 (29.9)	438 (70.1)		
Non-married	5 (6.2)	76 (93.8)		
BMI			41.869	< 0.001
Severely underweight/underweight	3 (9.4)	29 (90.6)		
Normal weight	44 (17.2)	212 (82.8)		
Overweight	78 (43.3)	102 (56.7)		
Obesity	67 (28.2)	171 (71.8)		
Self-related health			16.271	< 0.001
Self-related poor / fair health	27 (15.4)	148 (84.6)		
Self-related good health	165 (31.1)	366 (68.9)		

Table 4 presented the final logistic regression analysis, residence (OR = 0.552, 95% CI 0.365 to 0.833, *P* = 0.005), age (OR = 0.343, 95% CI 0.234 to 0.503, *P* < 0.001), marriage (OR = 0.227, 95% CI 0.087 to 0.592, *P* = 0.002), BMI (normal weight: OR = 1.782, 95% CI 1.124 to 2.826, *P* = 0.014; overweight: OR = 0.483, 95% CI 0.310 to 0.751, *P* = 0.001), and self-related health (OR = 2.461, 95% CI 1.517 to 3.994, *P* < 0.001) were all significantly associated with the presence of multiple geriatric syndromes in older adults.

**Table 4:** Logistic regression model of risk factors for multiple geriatric syndromes among older adults in the southwest of China.

Variable	B	S.E	Wald	OR	95% CI		*P* value
					Lower	Upper	
**Residence**							
Rural	ref						
Urban	− 0.595	0.210	7.993	0.552	0.365	0.833	**0.005**
**Age()**							
≥ 70 years old	ref						
60–69 years old	− 1.070	0.195	30.049	0.343	0.234	0.503	** < 0.001**
**Sex**							
Female	ref						
Male	− 0.155	0.201	0.596	0.857	0.578	1.269	0.440
Education level			4.535				0.104
High	ref						
Low	0.005	0.259	0.000	1.005	0.605	1.671	0.983
Middle	0.517	0.296	3.059	1.677	0.940	2.993	0.080
Marriage							
Non-married	ref						
Married	− 1.483	0.489	9.191	0.227	0.087	0.592	**0.002**
BMI			33.758				** < 0.001**
Obesity reference	ref						
Severely underweight/underweight	1.142	0.643	3.152	3.134	0.888	11.062	0.076
Normal weight	0.578	0.235	6.031	1.782	1.124	2.826	**0.014**
Overweight	− 0.729	0.226	10.413	0.483	0.310	0.751	**0.001**
**Self-related health**							
Good	ref						
Poor/fair	0.901	0.247	13.303	2.461	1.517	3.994	** < 0.001**
Constant	3.110	0.585	28.269	22.425			** < 0.001**

ref.: reference.

## Discussion

The purpose of the present study was to describe the prevalence of geriatric syndrome and multiple geriatric syndromes related factors among older adults in China.

Our findings indicated that several factors significantly associated with multiple geriatric syndromes in older adults, including residence, age, marital status, BMI, and self-related health.

In this study, we observed geriatric syndromes in 706 older adults in Chengdu, Southwest China. We found that 90.5% of older adults had at least one geriatric syndrome and 72.8% experienced multiple geriatric syndromes. The prevalence of specific geriatric syndromes varied significantly, from 58.5% for polypharmacy and 5.1% for fall. Our findings regarding frailty were consistent with previous research by Sanford^44^, which reported that 41.0% and 30.4% of 11,344 individuals ≥ 65 years old across Missouri met the screening criteria for prefrailty and frailty, respectively, and 28.1% screened positive for dementia. Another cross-sectional population-based study^45^, with a sample of 1,705 individuals aged 60 years old or older, between 2009 and 2010 in Brazil found that the prevalence of polypharmacy was 32%, which was lower than that in our study. The high prevalence of polypharmacy may attribute to that more than 1/4 older adults in china suffer from multiple chronic disease^46^.

In our study, we found that 514 older adults (72.8%) had multiple geriatric syndromes (≥ 2 geriatric syndromes), which was higher than the study by Yang^25^. Yang’s study found that 40.2% of community-dwelling older adults with ≥ 2 geriatric syndromes^25^. The discrepancy in the prevalence of multiple geriatric syndromes may reflect underlying regional and lifestyle factors that warrant further exploration. A growing number of studies have focused on somatic and mental geriatric syndromes. In Veizi’s study they found that the most common geriatric syndrome was incontinence (69%), followed by polypharmacy (60%) and depression (43%)^21^. A cross-sectional study conducted in four community clinics in Moscow reported that a decline in instrumental activity of daily living score was identified in 34.8% of the patients, a risk of malnutrition in 25.8%, probable cognitive impairment in 8.6%, and symptoms of depression in 36.2%^47^. Our findings are consistent with previous studies showing somatic and mental geriatric syndromes prevalence were high. These contributes to public health policy and practice to rationally allocate medical resources in China. Several suggestions are proposed in our study: first, the government should improve the layout of elderly medical resources and establish a sound elderly medical service network based on primary health centres, with hospitals as the core; second, health technicians in primary health centres should adopt the conventional model of elderly comprehensive evaluation, comorbidity management model, and multidisciplinary team work model, provide medical treatment for elderly, and maintain and restore their functional status to the greatest extent possible.

Poor/fair self-reported health can be associated with lower life satisfaction and mortality so as lead to multiple geriatric syndromes, clinicians must assess how the older adults subjectively view their own health^48^. Married older adults were less likely to suffer from multiple geriatric syndromes than those unmarried. Marriage provides several benefits, including a lower mortality risk, a source of self-esteem, a supportive relationship, shared household goods, and the opportunity to combined accumulated assets and wealth^49^. Individuals aged 60–69 years old were less likely to suffer from multiple geriatric syndromes than those aged 70 years old and older. Aging remains the main risk factor for multiple geriatric syndromes, and the association may be based on the physiological changes due to cellular senescence, chronic inflammation, and the imbalance in the cellular redox state^50^.

Interestingly, our findings indicated that older adults living in urban communities were less likely to experience multiple geriatric syndromes compared to those living in rural communities. Older adults living in rural communities are more likely to have multiple geriatric syndromes, possibly because food, water quality, and social care systems between rural and urban communities are different^51,52^. Furthermore, we observed that normal weight individuals were more prone to multiple geriatric syndromes compared to obese older adults, while overweight individuals were less likely to suffer from multiple geriatric syndromes compared to obese older adults. This finding may be attributed to the observation of frailty in older adults, with weight loss being a common component^53^. Thus, adequate nutritional status is a key point to consider when establishing preventive measures. It is high time for medical staff to strengthen early screening, intervention, and classification management of geriatric syndrome among older adults, particularly those who are older, unmarried, and reside in rural communities. Medical staff should utilize diverse methods and media to implement health educational programs for the elderly and their caregivers, focusing on topics such as nutritious diets, physical activity and fitness, mental health, and rational medication usage. This effort aims to foster a healthy lifestyle and enhance the overall health literacy of older adults. At the same time, a follow-up management for geriatric syndrome is important to provide care services throughout their life in older adults.

The current study aimed to provide valuable evidence for community nurses to prevent multiple geriatric syndromes among older adults. To achieve this, a large sample of 706 older adults from Southwest China was recruited. Additionally, two trained research nurses were used to minimize differences in subjective observation. While our study provides valuable insights, it is not without limitations. Firstly, the cross-sectional study prevents the establishment of causal relationships. Secondly, as the study was conducted in a single city in China, the results may not be generalized to other regions in the country. Given these limitations, further research is warranted to confirm these findings and their broader applicability.

## Conclusion

The findings of the current study indicated that geriatric syndromes, especially polypharmacy, malnutrition/at risk of malnutrition, multimorbidity and frail/prefrail, were common among older adults. Over one third older adults had somatic-mental disorders and more than 70% had multiple geriatric syndromes (≥ 2 geriatric syndromes). In conclusion, older adults who were older, lived in rural communities, were unmarried, had a normal BMI, and had poor or fair self-reported health were more likely to have multiple geriatric syndromes.

This study highlights that multiple geriatric syndromes are prevalent among community-dwelling older adults in China, and underscores the significance of certain demographic factors in their occurrence. However, further longitudinal studies are needed to establish the temporal relationship between multiple geriatric syndromes and these demographic factors. Additionally, exploring causal relationships and effective prevention strategies for geriatric syndrome is essential to improve the health and well-being of older adults.

## Data availability statement

Data are available upon reasonable request to the first author.
